# YKL-40 in serum: a promising biomarker of juvenile SLE and strongly correlated with disease duration

**DOI:** 10.1007/s11845-023-03545-w

**Published:** 2023-10-24

**Authors:** Asmaa A. Ali, Rasha N. Yousef, Mai S. Elsheikh, Abeer R. Salamah, Liang L. Wu, Alshaimaa R. Alnaggar, Noha M. Khalil, Mervat E. Behiry

**Affiliations:** 1 Department of Laboratory Medicine, School of Medicine, Jiangsu University, Zhenjiang, 212013 P. R. Zhenjiang, China; 2 Department of Clinical and Chemical Pathology, Medical Research and Clinical Studies Institute, National Research Center, Giza, Egypt; 3 Department of Complementary Medicine, Medical Research and Clinical Studies Institute, National Research Center, Giza, Egypt; 4 Department of Molecular Genetics and Enzymology, Human Genetics and Genomic Research Institute, National Research Center, Giza, Egypt; 5 Internal Medicine Department, Rheumatology and Clinical Immunology Unit, KasrAlainy School of Medicine, Cairo University, Cairo, Egypt

**Keywords:** Disease duration, Juvenile onset SLE, SLE, YKL-40

## Abstract

**Background:**

The biological function of YKL-40 is not well determined in different inflammatory and autoimmune diseases; however, some data highlighted its possible connection with disease activity.

**Aim:**

We investigated the diagnostic utility of serum YKL-40 in patients with SLE and examined its correlation with disease activity. Additionally, we examined any differences in serum YKL-40 levels between juvenile and adult SLE patients.

**Methods:**

We included 78 female patients with SLE and 42 controls. The level of YKL-40 in serum was measured by ELISA.

**Results:**

The serum YKL-40 level in SLE patients was significantly higher compared to the control group (9 (3) ng/mL vs. 5.5 (0.1) ng/mL; *p* < 0.001). YKL-40 showed excellent diagnostic utility with an AUC of 1 (*p* < 0.001) and a cutoff point of 5.6, providing sensitivity and specificity of 100%. YKL-40 was higher in adolescents and those with a positive family history of SLE (*p* = 0.01 for both) and positively correlated with disease duration (*r* = 0.45, *p* < 0.001). YKL-40 level was significantly higher in patients with photosensitivity, fever, vasculitis, blood disorders, positive anti-dsDNA, and APL ab (*p* < 0.05 for all). Conversely, patients with skin manifestations had a significantly lower YKL-40 (*p* = 0.004). In juvenile SLE, the AUC was 0.65 and a *p*-value of 0.01, and at a cutoff value of (8.7) ng/mL, the sensitivity and specificity were 72% and 60%, respectively.

**Conclusion:**

YKL-40 in serum could be a promising biomarker in patients with SLE, especially in adolescent-onset cases. It is independently influenced by disease duration, anemia, thrombocytopenia, positive anti-dsDNA, and APL ab features.

## Introduction

Chitinase-3-like 1 protein (YKL-40) is a member of the glycoside 18 families of chitinases. It is a heparin-and-chitin binding glycoprotein with a molecular weight of approximately 40-kDa and is coded by the CHI3L1 gene located on chromosome 1q31-q32 [1].

YKL-40 glycoprotein is secreted by inflammatory cells, such as neutrophils and macrophages [2, 3]. YKL-40 is associated with numerous physiological processes, for example, inflammation, angiogenesis, tissue fibrosis, cell proliferation, and tissue remodeling [4]. YKL-40 is also involved in various inflammatory responses, stimulating pro-inflammatory cytokines production (for example, IL-18, IL-6, and tumor necrosis factor alpha). These cytokines, in turn, can increase serum levels of YKL-40, creating a feedback mechanism [5].

YKL-40 mRNA expression in human monocytes is strongly induced by IFNγ and inhibited by dexamethasone and IL-4. Studies have shown that the pro-inflammatory hormones parathyroid hormone-related protein (PTHrP) and arg-vasopressin (AVP) have differential effects on YKL-40 secretion by cultured chondrocytes. Both PTHrP and AVP stimulate YKL-40 secretion from chondrocytes of rheumatoid arthritis patients but do not affect patients with osteoarthritis. Interestingly, AVP inhibits YKL-40 secretion from chondrocytes of healthy individuals, while PTHrP does not change YKL-40 levels [6].

YKL-40 has been shown to dose-dependently stimulate human connective tissue cells proliferation [7]. It has a key role in sensitization of antigen and IgE stimulation, antigen-induced T-helper 2 response, along with innate immune cells activation. While it may act as a fibroblast mitogen and activate numerous signaling pathways, though, no cell surface receptor for YKL-40 has been identified [8].

Recently, studies found that YKL-40 has an important role in several autoimmune diseases such as systemic lupus erythematosus, systemic sclerosis, rheumatoid arthritis, Behçet disease, and inflammatory bowel disease. In rheumatoid arthritis, not only is the level of YKL-40 increased in serum, but it is also positively correlated with disease activity [9, 10].

In systemic sclerosis, YKL-40 serum levels mainly correlate with joint and lung involvement [11], and digital articular deformities [12]. High levels of serum YKL-40 are also observed in patients with polymyositis and dermatomyositis, and these levels show a correlation with disease severity and activity, along with lung involvement [13–15].

Systemic lupus erythematosus (SLE) is an autoimmune disease that is associated with T cell over activation and autoantibodies overproduction [16]. Juvenile systemic lupus erythematosus (JSLE) is characterized by disease onset before the age of 18 and typically presents with a more severe disease phenotype, morbidity, and mortality than adult-onset SLE, often associated with a poorer prognosis. JSLE represents approximately 15–20% of all SLE cases. Elevated levels of YKL-40 in untreated children with juvenile idiopathic arthritis (JIA) prove the intra-articular synthesis of YKL-40 by macrophages, fibroblast-like synovial cells, chondrocytes, and neutrophils [17].

In SLE, YKL-40 has higher serum levels than healthy controls [18, 19]. However, its levels did not correlate with disease activity [18]. A previous study revealed that YKL-40 plasma levels are almost twice greater in patients with SLE than in controls [19].

In the current work, we aimed to study the role of serum YKL-40 in patients diagnosed with systemic lupus erythematosus (SLE) and to determine its correlation with disease activity. We also aimed to examine any differences in serum YKL-40 levels between juvenile and adult SLE patients.

## Patients and methods

### Study setting and design

A cross-sectional study that was conducted in the Medical research center and Department of internal medicine and Rheumatology; Kaser Alainy Educational Hospital.

### Study subjects

The study included 78 female patients with SLE fulfilling the European League Against Rheumatism and the American College of Rheumatology (EULAR/ACR) 2019 classification criteria [20] and 42 healthy age- and sex-matched control subjects were included in the study. The cases were enrolled from outpatient clinic from May 2022 to the end of October 2022. The main inclusion criteria were the age range of patients; hence, we enrolled only patients from 14 to 24 years. Other autoimmune diseases were excluded from the study besides patients with associated infection, or those admitted during disease exacerbation.

### Ethical approval

Written informed consent was obtained from all participants according to the Declaration of Helsinki, and the study protocol was approved by the ethics committee of the National Research Centre, the reference number is 16385. All patients were subjected to full medical history and clinical examination. SLE disease activity index 2000 (SLEDAI-2 K) was assessed [21].

The sample size was calculated using the Minitab program and based on the prevalence of SLE disease in Egypt, which was determined to be 11.3/per 100,000 female populations, with juvenile SLE accounting for 8.6% of all total SLE cases, as indicated by a recent study [22]. Assuming a type I error of 6%, a type II error of 20%, and a 90% confidence interval, the minimum sample size required to achieve 80% power was calculated to be 66.

### Methodology

#### YKL-40 measurement in serum

Venous blood samples were drawn in the morning. Serum specimens were obtained after the samples were centrifuged at 2500 × *g* for 10 min. Serum specimens for YKL-40 were frozen at − 80 °C until analysis. Serum YKL-40 level was determined with an enzyme immunoassay method using the commercially available test MicroVue YKL-40 (Quidel, San Diego, CA, USA) using streptavidin-coated microplate wells, a biotinylated-Fab monoclonal capture antibody, and an alkaline phosphatase-labelled polyclonal detection antibody. The intra-assay and inter-assay coefficients of variation were 6.0 and 6.6%, respectively. The assessments were calibrated by the calibrators inserted in the kit. All measurements were done in duplicate.

### Statistical analysis

The statistical analysis was done using Minitab 17.1.0.0 for windows (Minitab Inc., 2013, Pennsylvania, USA). Continuous data was presented as mean and SD and categorical data as number and (%). The normality of data was examined using Shapiro–Wilk test. Independent *t*-test was used for comparison between two groups of continuous data nature, Pearson correlation coefficient was used to estimate the liner relationship between numerical data, and receiver operating characteristic curve (ROC-curve) was used to examine the performance of YKL-40 in SLE as well as juvenile SLE; additionally, multiple linear regression with stepwise selection models had been used to estimate factors influencing the level of YKL-40 in SLE patients. All tests were two-sided, *p* considered significant if ≤ 0.05.

## Results

### Demographic characters

**Fig. 1:**
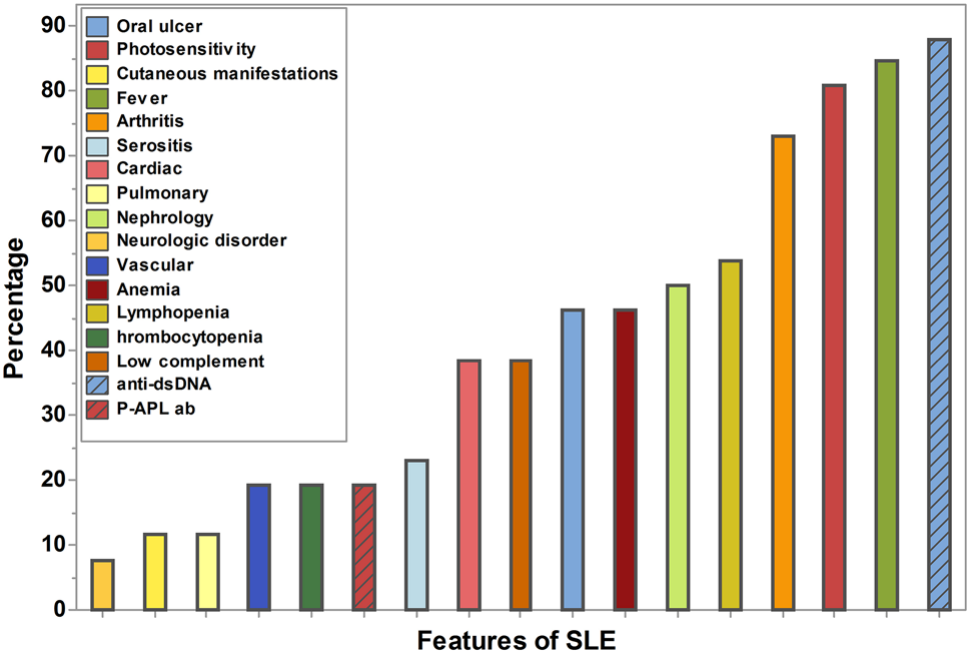
Abundance of SLE features

In Table 1, the mean age of the female SLE patients was 18 (SD = 3) years, with 42.31% of them being children (< 18 years), and the median duration of the disease was 22 (IQR = 12–36) months. The disease activity ranged from 0 to 17, and only 15.38% of cases were controlled. Figure 1 illustrates the abundance of SLE features, with positive anti-dsDNA, fever, photosensitivity, and arthritis being the most common (above 70%). In contrast, neurological, skin, and pulmonary manifestations were less common (less than 12%).

**Table 1:** Demographic characters of SLE cases (*n* = 78)

Factors	SLE	
Age, mean, SD	18.27	3.18
Age (< 18 Years), n,%	33	42.31
Family history (Yes), n, %	12	15.38
Disease duration (m), median, IQR	22	(12–36)
ESR, median, IQR	29.5	(22–35)
SLEDAI score, median, IQR 7	7	(5–11)
Treatment		
Cyclophosphamide	12	15.38
Azathioprine	30	40

The numerical data presented as mean and SD or median and IQR, the categorical data presented as number and percentage

*N* number, *SD* standard deviation, *IQR* interquartile range

### YKL-40 and SLE

**Fig. 2:**
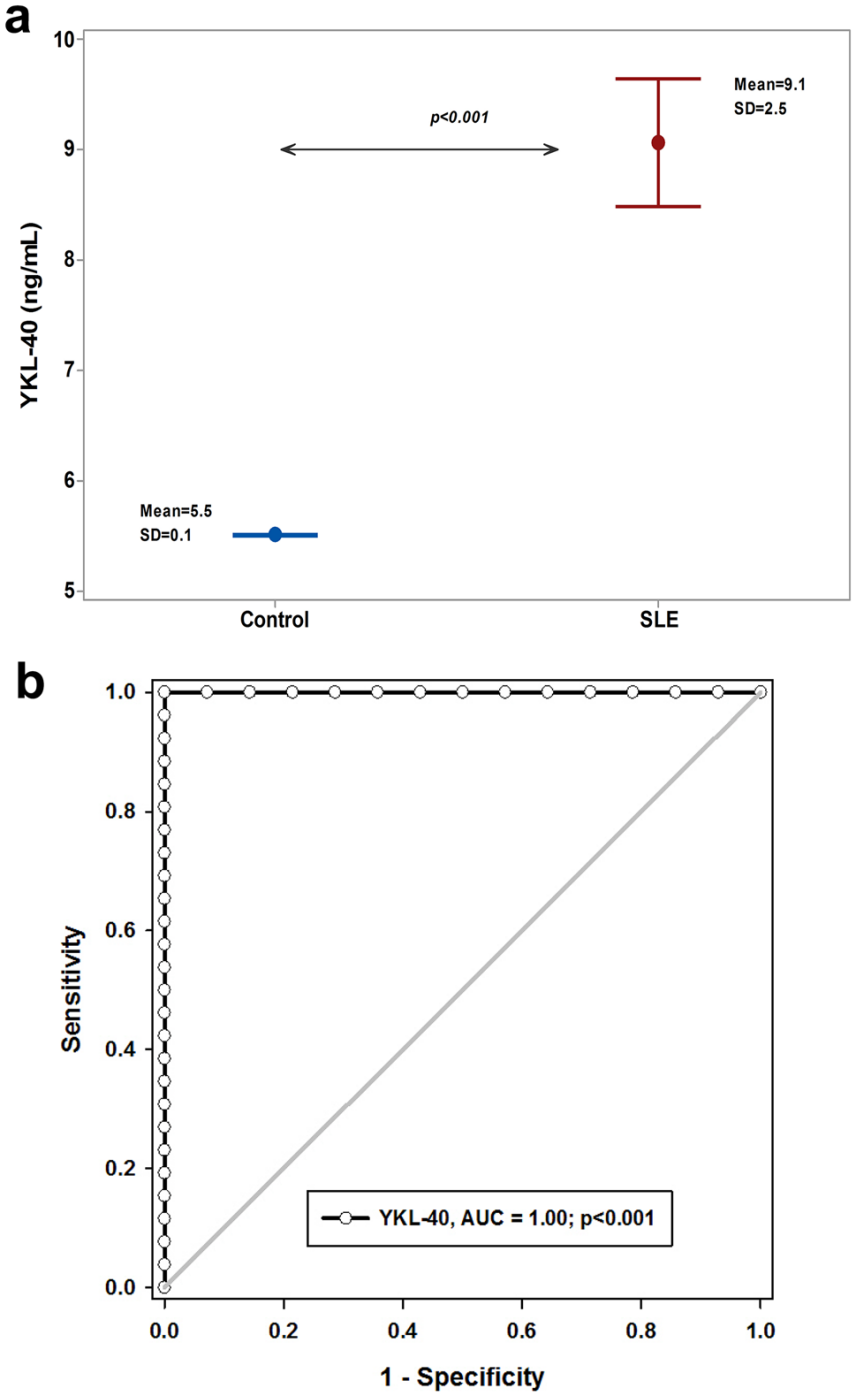
YKL-40 in patients with SLE: **a** Test of significant: independent *t*-test, **b** Diagnostic utility of YKL-40 in patients with SLE, AUC: area under curve, sensitivity and specificity were 100% at cutoff point above 5.6 (ng/mL), *p* < 0.05 considered significant

YKL-40 serum level in SLE patients was significantly higher compared to the control group (9 (3) ng/mL vs. 5.5 (0.1) ng/mL; *p* < 0.001). YKL-40 showed excellent diagnostic utility with an AUC of 1 (*p* < 0.001) and a cutoff point of 5.6, providing sensitivity and specificity of 100% (Fig. 2).

**Fig. 3:**
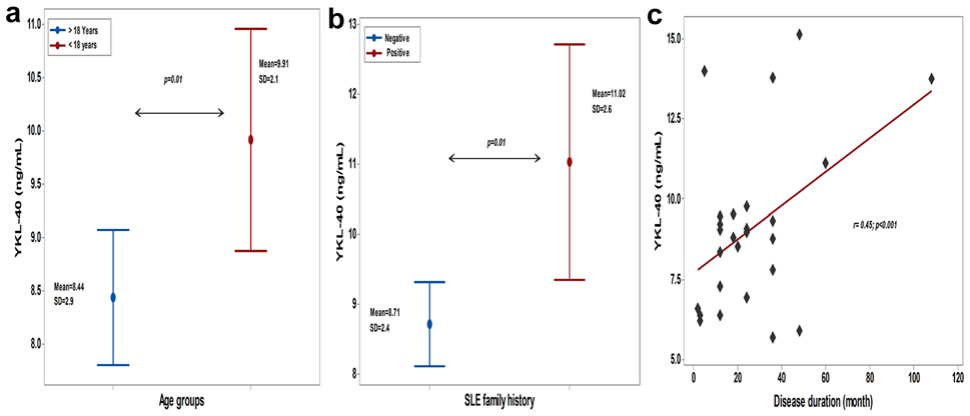
YKL-40 correlation with age group, family history and disease duration: **a** and **b** test of significance: independent *t*-test, **c** test of significance: Pearson correlation coefficient, the sign before “r” denote the direction of relationship, *p* < 0.05 considered significant

Furthermore, the level of serum YKL-40 varied significantly among SLE patients. It was higher in adolescents and those with a positive family history of SLE (*p* = 0.01 for both). The YKL-40 level was also positively correlated with disease duration (r = 0.45, *p* < 0.001) (Fig. 3).

Additionally, the YKL-40 level was significantly higher in patients with photosensitivity, fever, vasculitis, blood disorders (anemia, lymphopenia, and thrombocytopenia), positive anti dsDNA, and APL ab (*p* = 0.009, < 0.001, 0.006, 0.002, 0.012, < 0.001, 0.003, and 0.03, respectively).

Conversely, patients with skin manifestations had a significantly lower YKL-40 (*p* = 0.004). Finally, while cyclophosphamide treatment was associated with a significant increase in YKL-40 level (*p* = 0.01), no such correlation was observed with azathioprine treatment (Table 2).

**Table 2:** YKL-40 level in correlation with SLE features

**Factors**	**Total (*n* = 78)**	**YKL-40 (ng/mL)**		
	(n)	Mean	SD	*p*^*a*^
**Oral ulcer**	36	9.12	2.79	0.87
**Photosensitivity**	63	9.33	2.74	**0.009**
**Cutaneous manifestations**	9	7.32	1.45	**0.004**
**Fever**	66	9.39	2.62	**< 0.001**
**Arthritis**	57	9.09	2.66	0.87
**Serositis**	18	9.68	2.58	0.25
**Cardiac**	30	9.41	2.74	0.36
**Pulmonary**	9	8.59	1.02	0.26
**Nephrology**	39	8.6	2.3	0.11
**Neurologic disorder**	6	10.51	3.55	0.33
**Vascular**	15	11.23	3.16	**0.006**
**Anemia**	36	10.07	2.86	**0.002**
**Lymphopenia**	42	8.36	1.91	**0.012**
**Thrombocytopenia**	15	11.72	2.55	**< 0.001**
**Low complement**	30	9.05	1.96	0.95
**P- anti dsDNA**	66	9.35	2.63	**0.003**
**P-APL ab**	15	10.77	3.32	**0.03**
**Cyclophosphamide**	12	11.27	2.81	**0.01**
**Azathioprine**	30	9.5	2.21	0.21

The numerical data presented as mean and SD

*N* number, *SD* standard deviation

*P* < 0.05 considered significant

^a^independent *t*-test

**Fig. 4:**
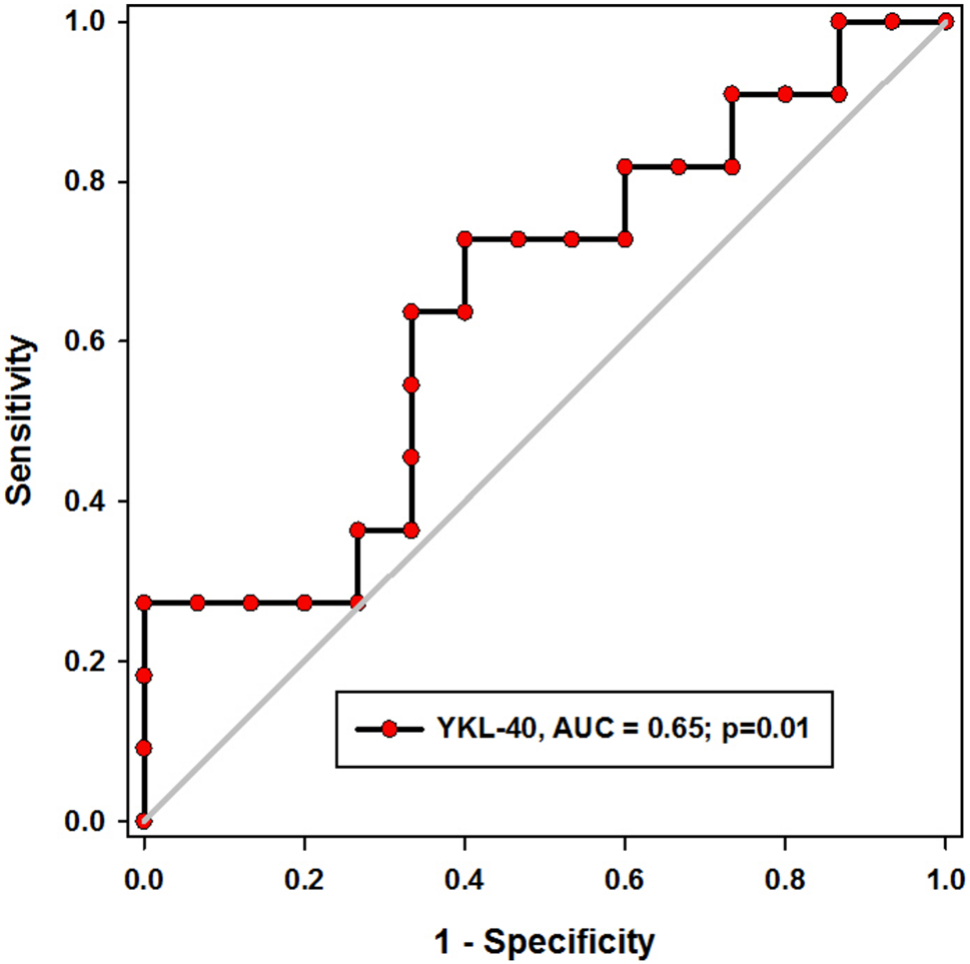
Utility of YKL-40 in juvenile SLE

The diagnostic potential of YKL-40 in distinguishing between juvenile and adult SLE was modest, with an AUC of 0.65 and a *p*-value of 0.01. At a cutoff value of (8.7) ng/mL, the sensitivity and specificity were 72% and 60%, respectively (Fig. 4).

Additionally, Table 3 revealed that several factors were strongly associated with elevated levels of YKL-40 in SLE patients, including longer disease duration, anemia, thrombocytopenia, positive anti-dsDNA and APL ab, all with a *p*-value < 0.05. Being juvenile was also identified as a significant factor promoting higher levels of YKL-40.

**Table 3:** Factors influencing the level of YKL-40 in SLE patients

Factors	CE	SE	*p*
Disease duration (m)	0.05	0.01	**< 0.001**
Age (< 18 years)	2.02	0.40	**< 0.001**
Anemia	1.21	0.36	**0.001**
Thrombocytopenia	1.57	0.49	**0.002**
P- anti-dsDNA	1.77	0.54	**0.002**
P-APL ab	1.25	0.47	**0.01**
Cyclophosphamide	0.97	0.52	0.07

The test of significance: Multiple linear Regressions with stepwise selection methods. The sign before coefficient represents the direction of relationship

*CE* coefficient of error, *SE* standard error

*p* < 0.05 considered significant

## Discussion

Macrophages are crucial in the development of SLE, as they may be polarized into either pro-inflammatory M1 or anti-inflammatory M2-like macrophages, depending on the microenvironment. The extent of polarization determines their functional properties, which can be either pro- or anti-inflammatory. This versatile property may have a possible role in the pathogenesis of autoimmune and inflammatory diseases [23].

Previous studies [24–26] have implicated M1-like macrophages in developing SLE due to their pro-inflammatory activity and ability to produce cytokines mediating autoimmune and chronic inflammatory diseases [27]. Clinical trials have also shown a positive correlation between the number of monocytes expressing M1 macrophage-like markers (CD163-CD14 +) in the peripheral blood of children with SLE and the severity of juvenile SLE [28]. M2-like macrophages have anti-inflammatory activity [24], though researchers found that these cells may have pro-inflammatory activity. Previous studies found that dysfunctional M2-like macrophages might contribute to the pathogenesis of SLE by producing excessive cytokines [29].

Additionally, defective M2-like macrophages cannot efficiently clear immune complexes (ICs), which can accumulate in various tissues and cause organ damage [23].

Studies have stated that macrophage activation syndrome (MAS) is more prevalent in patients with Juvenile SLE [30]. MAS is characterized by high fever, hepatosplenomegaly, hepatic dysfunction, pancytopenia, and clotting disorder. The precise role of macrophage polarization in SLE-MAS is not clear.

However, published data suggest that the uncontrolled release of pro-inflammatory cytokines by aberrantly activated macrophages and T lymphocytes is responsible for hematological and organ involvement in MAS [23]. Among these cytokines, TNF-α is of exact interest because it is more specific to SLE-MAS than to other inflammatory diseases [31].

The phenotype of YKL-40-producing macrophages differed from non-YKL-40-producing macrophages by increased production of TNFα and decreased expression of CD14 and HLA-DR. This finding suggests that production of YKL-40 is associated with macrophages with immunostimulatory functions but an impaired ability to recognize and present antigens or distinguish pathogen-associated molecular patterns [23].

Macrophages activation with IFN‐γ and LPS induces tenfold transcription of YKL‐40. Previous research by Bonneh-Barkay et al. demonstrated that exposure of astrocytes to macrophage culture medium (MCM) induced transcription of YKL-40 [32], which is consistent with previous in vivo results by the same researcher showing high expression of YKL-40 in reactive astrocytes in both acute and chronic neuro-inflammatory disorder, especially in areas close to inflammatory cells [33].

A one-day exposure of astrocytes to IL-1β was sufficient to induce transcription of YKL-40 for several consecutive days, which then declined to control levels 7 days later. Comparable results were observed in rat chondrocytes exposed to TNFα for 4 h, which showed raised YKL-40 mRNA and protein levels up to 72 h after cytokine removal [34].

In vivo, this temporary pattern of YKL-40 expression has also been observed in earlier studies of traumatic brain injury models (TBI) as the YKL-40 levels are significantly correlated with CSF levels of inflammatory cytokines, such as TNF-α and IL-1β, as well as with the inflammatory marker CRP [35]. This may explain our findings as the serum level of YKL-40 in SLE patients was significantly higher in adolescent than in adult. As JSLE is associated with greater disease activity, with regard to TBI and SLE, recent studies indicate that inflammatory and autoimmune processes are likely to be important components of the disease process leading to TBI, which subsequently resulted in loss of cognitive abilities, including dementia [36]. Additionally, the YKL-40 revealed significant positive correlation with disease duration.

In the current study, we investigated serum level of YKL-40 as a potential biomarker of SLE. We compared YKL-40 serum levels in samples from SLE patients with those from healthy controls. Our results showed an association between high serum YKL-40 levels and SLE; however, we found no correlation with disease activity. These findings suggest that YKL-40 may be a useful diagnostic marker for SLE, this is in concordance with Wcisło-Dziadecka et al. in 2009, who found that mean serum YKL-40 levels were almost twice as high in SLE patients in comparison to controls and also found no correlation between YKL-40 serum levels and SLE disease activity as measured by the SLEDAI [19].

In SLE patients, hematological disorders are frequently observed, including anemia, thrombocytopenia, leukopenia, lymphopenia, splenomegaly, and lymphadenopathy [37]. In the current study, the levels of serum YKL-40 were significantly elevated in SLE patients with blood disorders such as anemia, lymphopenia, and thrombocytopenia. These findings suggest that YKL-40 could be a biomarker for SLE patients’ hematological involvement. However, further research is required to determine the clinical utility of YKL-40 in this context. Anemia in SLE results from the upregulation of hepcidin, a protein that prevents iron from being incorporated into red blood cells for growth. Hepcidin seems to be controlled by inflammatory cytokines (principally IL6), which promotes its synthesis by activating signal transducer and activator of transcription 3. TNF-α, IFN-γ, and IL-1 interfere with iron homeostasis by increasing ferritin synthesis and decreasing cell-surface transferrin receptor concentration. As previously mentioned, YKL-40 stimulates the production of pro-inflammatory cytokines, such as IL-6, IL-18, and TNF-α, which can influence serum YKL-40 levels through feedback mechanism [5]. A study by den Broeder et al. revealed that TNF-α neutralization reduced the YKL-40 levels in RA patients, supporting the role of TNF-α in the regulation of YKL-40 levels [38].

The incidence of Lymphopenia in SLE patients is reported to be 20 to 75%, mostly involving regulatory T-cells. In patients with different subtypes of SLE, helper T cells are often reduced while the disease is still active [37]. A study by Kavanaugh et al. showed that HLA-DR4B1-Org3660l, a peptide derived from YKL-40, could suppress the T-cell response and stimulate immune tolerance in rheumatoid arthritis patient [39]. These findings suggest a potential therapeutic use of YKL-40-derived peptides in autoimmune diseases.

Twenty-five to 50% of SLE cases have mild thrombocytopenia, whereas severe thrombocytopenia is observed in nearly 10% of cases. Thrombocytopenia in SLE is caused mainly by immune thrombocytopenia. Although the pathophysiology is not fully understood, it may be because of the involvement of specific IgG antibodies formed by B lymphocytes against glycoproteins Ib/IX, IIb/IIIa, and Ia/IIa [37].

Antiphospholipid antibodies and other autoantibodies are identified in some patients with SLE. Phospholipid antibodies against cardiolipin, prothrombin, phosphatidylinositol and lupus anticoagulants are noticed in many SLE patients with thrombocytopenia. Membrane phospholipids that cross-react with cardiolipin antigen are exposed after cell affection and may stimulate the development of anti-cardiolipin antibodies [37]. This may explain why in our study, YKL-40 serum levels were significantly higher in SLE patients with antiphospholipid antibodies and thrombocytopenia. Contrary to our findings, YKL-40 did not correlate with thrombocytopenia in a research done by Outinen et al. [40]. In the current study, individuals with skin manifestation showed significant lower level of YKL-40, this is not in agreement with a previous Japanese study that stated elevated levels of YKL-40 in Japanese patients with psoriasis and suggested that YKL-40 serum levels can act as a valuable biomarker for detecting the severity of dermatological lesions in psoriasis patients [41].

YKL-40 serum levels were higher in SLE patients with photosensitivity presentation, fever, and vasculitis; this may suggest that serum YKL-40 may play a role in the inflammatory process of SLE during disease activity and may serve as a valuable laboratory test to detect SLE activity. This is in accordance with Gamal El-Agamy et al. results [42].

## Conclusion

The diagnostic potential of serum YKL-40 in SLE was excellent, especially in juvenile cases. It was higher in adolescents and those with a positive family history of SLE and correlated directly with disease duration. Additionally, anemia, thrombocytopenia, positive anti-dsDNA, and antiphospholipid antibodies significantly promote the higher level of YKL-40 in patients with SLE.

## Data Availability

The authors confirm that the data supporting the findings of this study are available within the article.

## References

[1] Tizaoui K, Yang JW, Lee KH (2022). The role of YKL-40 in the pathogenesis of autoimmune diseases: a comprehensive review. Int J Biol Sci.

[2] Hakala BE, White C, Recklies AD (1993). Human cartilage gp-39, a major secretory product of articular chondrocytes and synovial cells, is a mammalian member of a chitinase protein family. J Biol Chem.

[3] de Ceuninck F, Gaufillier S, Bonnaud A (2001). YKL-40 (Cartilage gp-39) induces proliferative events in cultured chondrocytes and synoviocytes and increases glycosaminoglycan synthesis in chondrocytes. Biochem Biophys Res Commun.

[4] Lee CG, da Silva CA, dela Cruz CS (2011). Role of chitin and chitinase/chitinase-like proteins in inflammation, tissue remodeling, and injury. Annu Rev Physiol.

[5] Carboni RCDS, Behrens-Pinto GL, Shinjo SK (2021). High YKL-40 serum levels and its expression in the muscle tissues of patients with antisynthetase syndrome. Adv Rheumatol.

[6] Kzhyshkowska J, Gratchev A, Goerdt S (2007). Human chitinases and chitinase-like proteins as indicators for inflammation and cancer. Biomark Insights.

[7] Baeten D, Boots AMH, Steenbakkers PGA (2000). Human cartilage gp-39, CD16+ monocytes in peripheral blood and synovium: correlation with joint destruction in rheumatoid arthritis. Arthritis Rheum.

[8] Coffman FD (2008). Chitinase 3-Like-1 (CHI3L1): a putative disease marker at the interface of proteomics and glycomics. Crit Rev Clin Lab Sci.

[9] Väänänen T, Vuolteenaho K, Kautiainen H et al (2017) Glycoprotein YKL-40: a potential biomarker of disease activity in rheumatoid arthritis during intensive treatment with csDMARDs and infliximab. Evidence from the randomised controlled NEO-RACo trial. PLoS One 12(8) 10.1371/journal.pone.0183294 PMC5571914 28841649

[10] Jafari-Nakhjavani MR, Ghorbanihaghjo A, Bagherzadeh-Nobari B (2019). Serum YKL-40 levels and disease characteristics in patients with rheumatoid arthritis. Caspian J Intern Med.

[11] La Montagna G, D’Angelo S, Valentini G (2003). Cross-sectional evaluation of YKL-40 serum. J Rheumatol.

[12] Nordenbæk C, Johansen JS, Halberg P (2005). High serum levels of YKL-40 in patients with systemic sclerosis are associated with pulmonary involvement. Scand J Rheumatol.

[13] Hozumi H, Fujisawa T, Enomoto N (2017). Clinical utility of YKL-40 in polymyositis/dermatomyositis-associated interstitial lung disease. J Rheumatol.

[14] Gao MZ, Wei YY, Xu QW (2019). Elevated serum YKL-40 correlates with clinical characteristics in patients with polymyositis or dermatomyositis. Ann Clin Biochem.

[15] Jiang L, Wang Y, Peng Q (2019). Serum YKL-40 level is associated with severity of interstitial lung disease and poor prognosis in dermatomyositis with anti-MDA5 antibody. Clin Rheumatol.

[16] AlDeen HG, Ramadan A, Awadallah E (2022). Patterns of microRNAs 142–3p, 106a, 17 and 20a expression in patients with systemic lupus erythematosus. Egypt Rheumatol.

[17] Greenan-Barrett J, Doolan G, Shah D et al (2021) Biomarkers associated with organ-specific involvement in juvenile systemic lupus erythematosus. Int J Mol Sci 22(14) 10.3390/ijms22147619 PMC8306911 34299237

[18] Peltomaa R, Paimela L, Harvey S (2001). Increased level of YKL-40 in sera from patients with early rheumatoid arthritis: a new marker for disease activity. Rheumatol Int.

[19] Wcisło-Dziadecka D, Kotulska A, Brzezińska-Wcisło L (2009). Serum human cartilage glycoprotein-39 levels in patients with systemic lupus erythematosus. Pol Arch Med Wewn.

[20] Aringer M, Costenbader K, Daikh D, Brinks R, Mosca M, Ramsey-Goldman R (2019). European League Against Rheumatism/American College of Rheumatology classification criteria for systemic lupus erythematosus. Arthritis Rheumatol.

[21] Gladman DD, Goldsmith CH, Urowitz MB (2000). The Systemic Lupus International Collaborating Clinics/American College of Rheumatology (SLICC/ACR) damage index for systemic lupus erythematosus international comparison. J Rheumatol.

[22] Gheita TA, Noor RA, Abualfadl E (2021). Adult systemic lupus erythematosus in Egypt: the nation-wide spectrum of 3661 patients and world-wide standpoint. Lupus.

[23] Ahamada MM, Jia Y, Wu X (2021) Macrophage polarization and plasticity in systemic lupus erythematosus. Front Immunol 12 10.3389/fimmu.2021.734008 PMC8721097 34987500

[24] Funes SC, Rios M, Escobar-Vera J, Kalergis AM (2018). Implications of macrophage polarization in autoimmunity. Immunology.

[25] Labonte AC, Kegerreis B, Geraci NS et al (2018) Identification of alterations in macrophage activation associated with disease activity in systemic lupus erythematosus. PLoS One 13(12) 10.1371/journal.pone.0208132 PMC6298676 30562343

[26] Orme J, Mohan C (2012). Macrophage subpopulations in systemic lupus erythematosus. Discov Med.

[27] Cuda CM, Pope RM, Perlman H (2016). The inflammatory role of phagocyte apoptotic pathways in rheumatic diseases. Nat Rev Rheumatol.

[28] Niu XL, Feng D, Hao S et al (2019) The significance of M1/M2 macrophage-like monocytes in children with systemic lupus erythematosus. Eur J Inflamm 17

[29] Ma C, Xia Y, Yang Q, Zhao Y (2019). The contribution of macrophages to systemic lupus erythematosus. Clin Immunol.

[30] Poudel P, Swe T, Rayancha S (2018). A rare case of macrophage activation syndrome presenting as the first manifestation of systemic lupus erythematosus. J Investig Med High Impact Case Rep.

[31] Mizuta M, Shimizu M, Irabu H (2021). Comparison of serum cytokine profiles in macrophage activation syndrome complicating different background rheumatic diseases in children. Rheumatology (United Kingdom).

[32] Bonneh-Barkay D, Bissel SJ, Kofler J (2012). Astrocyte and macrophage regulation of YKL-40 expression and cellular response in neuroinflammation. Brain Pathol.

[33] Bonneh-Barkay D, Wang G, Starkey A (2010). In vivo CHI3L1 (YKL-40) expression in astrocytes in acute and chronic neurological diseases. J Neuroinflammation.

[34] Recklies AD, Ling H, White C, Bernier SM (2005). Inflammatory cytokines induce production of CHI3L1 by articular chondrocytes. J Biol Chem.

[35] Bonneh-Barkay D, Zagadailov P, Zou H (2010). YKL-40 expression in traumatic brain injury: an initial analysis. J Neurotrauma.

[36] Shively SB, Wannamaker BB, Willis AM (2021). Traumatic brain injury and autoimmune disease. Front Neurol.

[37] Santacruz JC, Mantilla MJ, Rueda I (2022). A practical perspective of the hematologic manifestations of systemic lupus erythematosus. Cureus.

[38] den Broeder AA, Joosten LAB, Saxne T (2002). Long term anti-tumour necrosis factor alpha monotherapy in rheumatoid arthritis: effect on radiological course and prognostic value of markers of cartilage turnover and endothelial activation. Ann Rheum Dis.

[39] Kavanaugh A, Genovese M, Baughman J (2003). Allele and antigen-specific treatment of rheumatoid arthritis: a double blind, placebo controlled phase 1 trial. J Rheumatol.

[40] Outinen TK, Mantula P, Jaatinen P et al (2019) Glycoprotein YKL-40 is elevated and predicts disease severity in Puumala hantavirus infection. Viruses 11(9) 10.3390/v11090767 PMC6784349 31438470

[41] Imai Y, Aochi S, Iwatsuki K (2013). YKL-40 is a serum biomarker reflecting the severity of cutaneous lesions in psoriatic arthritis. J Dermatol.

[42] Gamal El-Agamy A et al (2023) Assessment of serum level of chitinase-3-like protein-1 (Ykl-40) in systemic lupus erythematosus patients and its relation to disease activity. J Adv Med Med Res 28–37

